# How to support adults with anorexia nervosa and autism: Qualitative study of clinical pathway case series

**DOI:** 10.3389/fpsyt.2022.1016287

**Published:** 2022-11-11

**Authors:** Zhuo Li, Chloe Hutchings-Hay, Sarah Byford, Kate Tchanturia

**Affiliations:** ^1^Department of Psychological Medicine, Institute of Psychiatry, Psychology, and Neuroscience, King's College London, London, United Kingdom; ^2^National Eating Disorders Service, South London and Maudsley NHS Foundation Trust, London, United Kingdom; ^3^King's Health Economics, Department of Health Service and Population Research, Institute of Psychiatry, Psychology, and Neuroscience, King's College London, London, United Kingdom; ^4^Tbilisi State Medical University, Psychological Set Research and Correction Center, Tbilisi, Georgia

**Keywords:** eating disorder, autism, comorbidity, treatment, adaptation

## Abstract

**Introduction:**

Previous research has explored the overlapping presentation between autism and eating disorders (ED). This study aims to summarize the clinical challenges associated with co-occurring autism and anorexia nervosa (AN) based on clinicians' case notes and minutes from case discussions, to understand how to better support people with the comorbidity.

**Method:**

Thematic analysis was conducted on de-identified notes on 20 cases with AN and autistic characteristics and minutes from case discussions. Themes relevant to clinical challenges in supporting those with the comorbidity were identified, and a thematic map was produced to visually represent the results.

**Results:**

The key challenges faced by clinicians when treating patients with AN and autism included: communication difficulties, maintaining boundaries, autism screening, presence of other comorbidities, sensory difficulties, atypical presentation of eating difficulties, cognitive rigidity, and emotional difficulties. Adaptations to resolve some of these difficulties included exposure-based food experiments, keeping a record of patients' self-reported communication preferences, individual-level modification of communication style, and providing tools for patients to identify emotions.

**Conclusions and implications:**

Further exploration to establish the effectiveness of the adaptations is warranted. Furthermore, tools for differentiating between ED, autism and other comorbidities are needed to help clinicians clarify the cause of a presenting symptom, and help them to best support and maintain boundaries with patients.

## Introduction

Anorexia nervosa (AN) is an eating disorder (ED) associated with the highest mortality rate among all psychiatric disorders (1) and has an average prevalence rate of 0.3% among young women (2). It is characterized by an intense fear of gaining weight, behaviors interfering with weight gain and a distorted body image. Other types of ED include bulimia nervosa (BN), characterized by binging and purging behaviors, and binge-eating disorder (BED), characterized by recurrent episodes of binge eating without compensatory behaviors. They are relatively less researched than AN but are more common in the population with prevalence rates of at least 1% (3, 4).

People with ED commonly present with psychiatric and medical comorbidities, such as anxiety, OCD, substance use and personality disorders (5). In particular, the overlap between AN and autism has been more actively researched in recent years, as summarized in a framework by Kinnaird and Tchanturia (6). Similarities between the two conditions include dietary restriction and food selectivity (7, 8), difficulties in cognitive flexibility (9), social anhedonia (10, 11), and strong interests in and preoccupation with specific topics (12, 13). The estimated prevalence of autism symptomatology in ED populations ranges from 8 to 37% (14–17), and individuals with co-occurring ED and autism are at risk of poorer treatment outcomes (19, 20). Overall, these findings highlight the need for individualized ED treatment adapted to the needs of autistic individuals.

The Pathway for Eating disorders and Autism developed from Clinical Experience (the PEACE pathway; https://www.peacepathway.org/) was developed and implemented in the South London and Maudsley (SLaM) NHS Foundation Trust Adult Eating Disorders Service with the aim of improving care for patients with co-occurring ED and autism (21, 22). It is, to our knowledge, the first systemic attempt to adapt an ED service for this comorbidity. Since the pathway's instigation in 2019, adaptations have been introduced relating to clinician training, psychoeducation resources (newly developed for patients), patient screening, treatment environment (to make it more sensory-friendly) and food menus.

As part of the implementation of the PEACE pathway from 2019 to 2022, weekly team meetings [known as ‘PEACE huddles'; (23)] have been held to discuss cases with the comorbidity, associated practical challenges and possible treatment adaptations. These discussions fed into further service adaptations supporting the continuous development of the PEACE pathway over time.

The aim of this study was to present a synthesis of clinical challenges associated with both autism and anorexia nervosa (AN) based on a review of the case notes and minutes from the huddle discussions, and to outline the team's approach to the subsequent adaptation of treatment. As a considerable body of research has already covered autistic features in AN as well as patients' experience accessing support (6, 18, 24, 25), it is hoped that this study will present the clinical reality faced by clinicians trying to individualize care for those with the comorbidity, and thereby inform decision-making and treatment adaptations for this population.

## Methods

### Study design

This study reports the results of a review of clinical case notes and team meeting minutes relating to patients with co-morbid ED and autism. The study was part of a service quality improvement project and permission to audit patient data was obtained from the Clinical Governance and Audit Committee in South London and Maudsley NHS Trust (032019) in April 2019. In accordance with the institutional requirements written consent from the participants was not required. All clinical notes were fully anonymised to protect patient privacy.

### Setting and sample

As part of the PEACE pathway implementation process at the SLaM Adult Eating Disorders Service (including inpatient and day services) between September 2019 and March 2022, clinicians attended regular PEACE huddle meetings to discuss select patient cases with complex presentation. The “PEACE huddles”, which were utilized as group supervision, provided attending clinicians with an opportunity to share thoughts and challenges about patients with autism and develop consistency in treatment implementation. Cases were discussed in huddles if they either had a previous diagnosis of autism or presented with autistic characteristics, and their treatment was considered challenging by the care team (e.g., atypical eating difficulties due to autism). All case notes were de-identified before they were shared among the team to aid discussion. Minutes from the discussion, which included suggestions for adaptations and feedback on what was helpful for the cases, were also circulated among the team after the huddles.

The de-identified clinical notes contained clinician-written case management notes, progress and updates, nursing notes, summary of challenges and exploration of autistic characteristics. At the ED service where the PEACE pathway was implemented, autistic characteristics were routinely explored for all patients admitted to the ED service using autism screening tools. Screening primarily involved application of the Autism Spectrum Quotient short version (AQ-10) (25, 26). Where deemed necessary by the care team, autistic characteristics were further explored using the Autism Diagnostic Observation Schedule Module 4 [ADOS; (27)] and/or the Social Responsiveness Scale, Second Edition [SRS-2; (28)], to provide more information for the care team and to guide treatment adaptation. In this study, both patients with a formal diagnosis of autism and those presenting with autistic characteristics will be referred to as individuals with “autism comorbidity” or “people with autism.”

### Data

This study collected data from de-identified clinical notes and minutes from the PEACE pathway team meetings, as both sources of information referred to treatment challenges, potential treatment adaptations to meet these challenges and feedback on adaptations that had been helpful. Thematic analysis (29, 30) was used to analyse clinicians' notes and minutes of the PEACE huddles to identify clinical challenges and adaptations in supporting adults with EDs and autism. ZL first read all case notes and minutes repeatedly to inductively generate and refine potential codes. Coded data were then analyzed to identify themes and subthemes relevant to clinical challenges and treatment adaptations in supporting those with the comorbidity, and a thematic map was developed to represent the themes and subthemes in a visual format. CH independently reviewed and checked the thematic map against the case notes. The final thematic map was reviewed and finalized in consultation with the principal author KT.

## Results

### Demographic characteristics

In total, 34 cases were discussed in the PEACE huddles. Thirteen cases were consultations by teams from other ED services and therefore excluded from analysis. One case discussion focused on scoring of the autism screening tools rather than patient presentation and was therefore excluded from the study. Table 1 shows the demographic characteristics of the remaining 20 cases that were included in the study.

**Table 1:** Summary of demographic information.

	**Cases (*n* = 20)**
**Gender, *n* (%)**	
- Female	16 (80%)
- Male	4 (20%)
**Ethnicity, *n* (%)**	
- White British	17 (85%)
- White Other	1 (5%)
- Black African	1 (5%)
- Asian	1 (5%)
**Age, mean (SD)**	26 (10.7)
**ED diagnosis, *n* (%)**	
- AN restrictive subtype	13 (65%)
- Atypical AN	5 (25%)
- AN binge-purge subtype	2 (10%)
**Number of co-morbidities (other than autism), mean (SD)**	1.85 (1.2)

The majority of cases were female (*n* = 16, 80%) and the mean age at contact with the service was 26 years (SD = 10.7, range 19–68). Half of the cases had a formal diagnosis of autism prior to contact with the ED service (*n* = 10, 50%), whilst the other half were flagged up by the AQ-10 or ADOS-2 as having high autistic characteristics and recommended to receive formal assessment at a specialist service (*n* = 10, 50%). In addition to autism or autistic characteristics, further co-morbidities were reported, the most common of which was generalized anxiety disorder (GAD; *n* = 10, 50%), obsessive-compulsive disorder (OCD; *n* = 8, 40%) and depression (*n* = 8, 40%). Other common co-morbidities included emotionally unstable personality disorder (EUPD; *n* = 2, 10%) and attention deficit hyperactivity disorder (ADHD; *n* = 2, 10%).

### Clinical challenges with meeting the needs of adults with AN and autism

**Figure 1:**
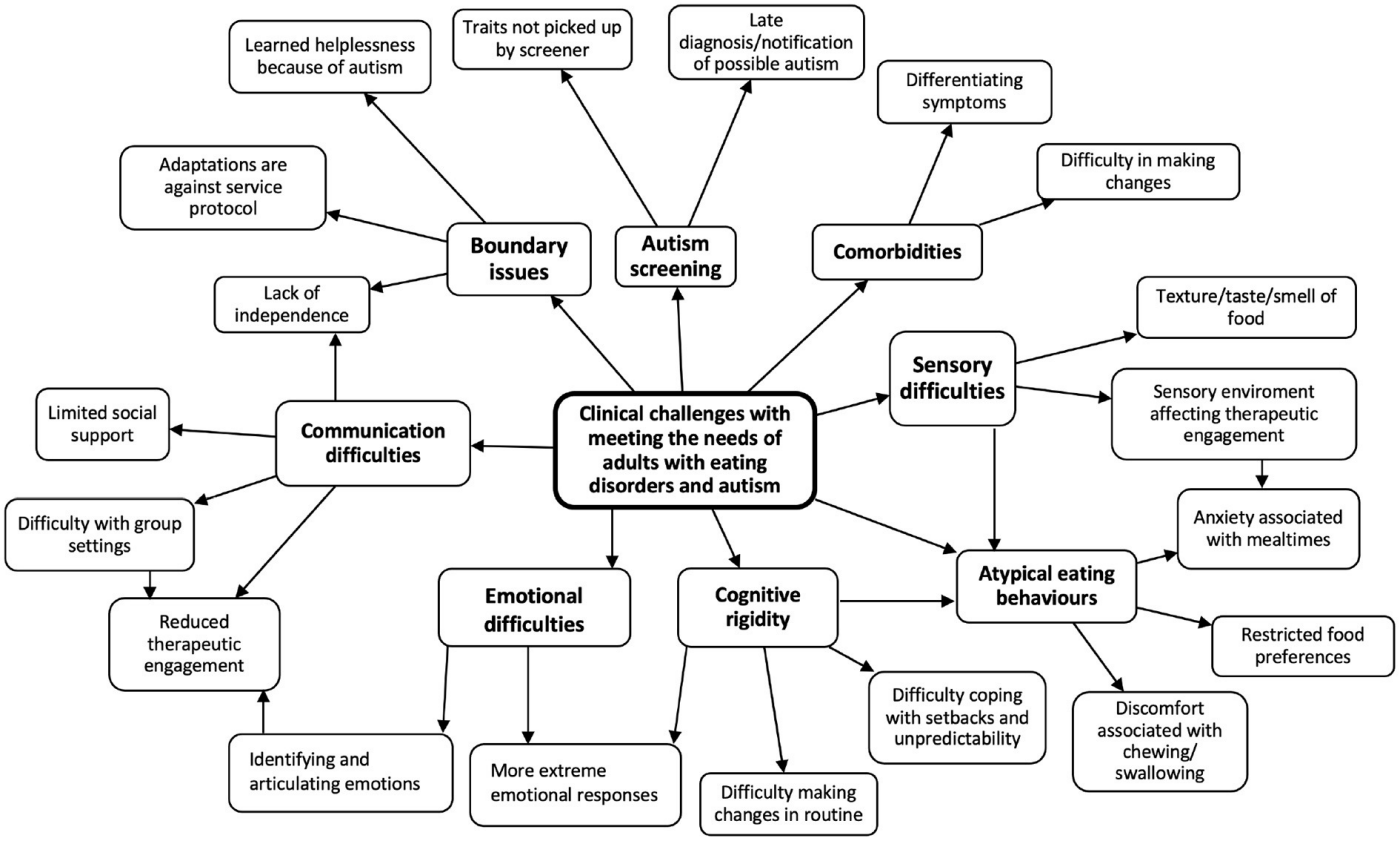
Thematic map: clinical challenges with meeting the needs of adults with eating disorders and autism.

Figure 1 shows the main themes that emerged from analysis of the case notes and meeting minutes. Subthemes that are relevant and connected to each other (e.g. Autistic traits not being picked up by the screener, Late diagnosis of autism) are categorized under a broader key theme (e.g. Autism screening), which is visualized in Figure 1. In total, eight key themes relevant to the research question were identified: communication difficulties, boundary issues, issues related to autism screening, presence of comorbidities, sensory difficulties, atypical eating behaviors, cognitive rigidity, and emotional difficulties.

#### Communication difficulties

Communication difficulties were highlighted in most cases, with severity ranging from mild difficulties in articulating thoughts to selective mutism. Patients also had issues with open questioning and found it hard to answer broad and open questions like: “how can I help?”. This affected patients' therapeutic engagement and posed challenges for the care team.

“[The patient] struggles with trying to explain what [the patient] means, [and with] verbalizing ED… Difficult knowing if the patient understands, there is lots of nodding and it seems fairly superficial at times.” (Case 20)

“Communication has been a struggle. Some meetings may have some verbal input from [the patient], but this is rare.” (Case 12)

The clinical team tried different ways of adapting care to support patients with communication difficulties, including individual-level modification of communication style and use of conversation cards and other resources (diagrams or art) to understand patients' preferred ways of communication. The team also asked patients to fill in the communication passport, which is a one-page self-report document including a patient's preferred way to communicate, their sensory needs, their dislikes and their special interests and strengths. The passport was then shared with the wider care team to ensure team awareness and consistency.

“Multiple choice is easier than ‘what do you need/how can I help'.” (Case 14)

“Continue using visual aids (which [the patient] found helpful so far).” (Case 4)

“Suggest revisit the communication passport; suggest written communication (patient is very good at writing letters).” (Case 15)

Communication difficulties also affected group participation, as some patients felt anxious about speaking in front of others and found the group therapy setting overwhelming. This affected both online groups and groups in person.

“[the patient] would often voice that [the patient] is quite anxious to speak in front of everyone. There are times where staff has noticed [the patient] has ‘checked out' from the group and looks disengaged by staring out in front of [the patient].” (Case 4)

“[the patient's] engagement in [online] groups was minimal and would not look at the screen as a way of avoiding eye contact.” (Case 19)

Looking for ways to encourage group participation, clinicians noticed that sometimes patients would only share information on direct questioning and therefore tried to encourage their participation by inviting them to contribute. For online groups, patients found it easier to be able to join with audio only.

“What worked well: inviting [the patient] to contribute and share [the patient's] views was helpful in groups” (Case 19)

“Voice-only session with the family is working well. Past cases also mentioned that audio-only sessions allow more space for patients to process what others are saying. Can be a useful adaptation for patients with autistic characteristics.” (Case 8)

Social isolation from peers was reported in all cases, within and outside treatment settings. This was sometimes accompanied by over-dependence on family carers and clinical staff, which was identified to be a major barrier to independent living and returning to the community after treatment completion.

“[The patient] can find it really difficult to make decisions, [the patient] asks for mum's support with certain things e.g. which therapy [the patient] should do/what to pick from the menu.” (Case 14)

#### Challenges in maintaining boundaries

Boundary issues were described in the majority of cases, with patients described as becoming over-dependent on therapeutic relationships, clinical teams having to adapt treatment in a way that was sometimes against ward protocol (e.g. allowing patients to touch or smell food without eating it, or to have headphones on during dinner time instead of social eating), and patients refusing change or treatment owing to a ‘learned helplessness' mindset about autism (i.e., insisting that their autism means that they are not capable of making changes essential to recovery and independent living). These boundary issues would often leave the clinical team with the difficult decision of whether to accommodate some of the autism-related difficulties or further encourage changes in the recovery journey from ED.

“[The patient's] sensory needs sometimes are in conflict with the ward protocol and other patients' needs…Team can struggle with when to accommodate and when to encourage for change.” (Case 15)

“Difficult to manage boundaries with [the patient]; Need to limit the number of adaptations which can be agreed.” (Case 6)

Compromises were often made to meet patients in the middle. However, when over-accommodation could risk impeding patients' recovery from their ED, clinicians would try to limit the number of adaptations that can be agreed and challenge patients' mindset with transparent, goal-oriented conversations to encourage changes.

“Challenge [patient's] mindset about ASD: positive mindset to manage and work on sensory sensitivities and other challenges autism brings, instead of a learned helplessness mindset (e.g., I have autism, I'm never going to be able to). Start by exploring strengths and gifts.” (Case 15)

#### Screening of potential autism

Autism screening brought further challenges for clinicians within the ED service. In all cases concerned, the AQ-10 (26) was used as a pragmatic short screener for potential autism, sometimes accompanied by an ADOS Module 4 (27) interview when a qualified ADOS-trained member of the team was available, or by an SRS-2 self-report questionnaire when an ADOS-trained interviewer was not available. However, some patients scored below the threshold on the AQ-10 despite their strong presentation of autism or already having a formal diagnosis. Furthermore, the majority of patients were not aware of their potential presentation of autism prior to the screener and had limited knowledge about autism. Therefore, informing patients and their families about a positive result on the screening tools was often met with surprise, causing anxiety for the patient and their families and for the clinical team member involved.

“…the results of the ADOS-2 created some anxiety for [the patient] and parents – they were left with questions needing a forum in which to raise them. Psychologist anxiety about leaving patient to process the report feedback and how best to support them.” (Case 7)

In their reflection, clinicians noted the need for more autism-related psychoeducation and training, particularly on normalizing autism and feeding back autism screening results to families.

“[R]eflections and what we learned included: how little people know about autism and the need for more psychoeducation; importance of being open with patients and families that we are trying to learn about the comorbidity; … identify patients' strengths and work with this; need for a learning training session on feeding back ADOS results to patients and carers and to normalize A[utism] S[pectrum] C[ondition].” (Case 7)

#### Comorbidities

Case notes and meeting minutes also documented the care team spending considerable time helping patients distinguish between problems caused by different comorbidities, such as between rigidity around food caused by autism and inflexible mealtime routines driven by obsessive-compulsive disorder (OCD), or between food avoidance caused by AN cognitions and sensory avoidance driven by autism. This was challenging because the cases presented with a variety of complex comorbidities (Table 1) and symptoms were often intertwined, sometimes fueling one another, making changes and recovery even more difficult.

“Fairly clear on what is AN vs. autism/OCD but it is harder to differentiate Autism and OCD due to the common factor of rigidity to routines etc.” (Case 16)

“Comorbidities predate ED and are intertwined with it. [OCD] symptoms … daily focused on fear of being ill. … restricting food because [the patient] is worried about being ill. …Autism makes routines even more rigid.” (Case 18)

Differentiating between behaviors caused by ED and other comorbidities is nevertheless important in establishing focus for treatment. Clinicians would work with patients collaboratively to differentiate between specific eating-related behaviors and identify those rooted in ED that required intervention.

“Using napkins to wipe hands is ED/sensory related, does not like the feeling of food on fingers. …Not completing meals based on OCD obsession. …ASC related – needing foods to be a specific ‘right temperature'.” (Case 6)

#### Sensory difficulties

Sensory difficulties also made it more difficult for clinicians to treat patients with the comorbidity, particularly at mealtimes. These sensory difficulties included sensitivity to texture, taste, or smell of certain foods on the menu, and sensory overload due to environmental factors that affected therapeutic engagement, such as distraction by the noise or brightness of the surroundings.

“Very sensitive to noise and lights. Describes [themselves] as having increased interoceptive awareness and [the patient] experiences lots of physical pain associated with this.” (Case 9)

“[The patient dislikes] flashing lights, loud noises, sudden noises such as clapping.” (Case 20)

“Hypersensitive to human sounds especially chewing food.” (Case 14)

Clinicians reported that cases with sensory sensitivities found attending workshops on sensory wellbeing psychoeducation helpful. Sensory items and low stimulus quiet areas were made available for patient use. Clinicians also adapted the environment of individual therapy sessions, checking in with patients in the beginning of the sessions to confirm if they felt comfortable in the environment.

“Attended the sensory wellbeing workshop and was really engaged with the content, and was able to complete the sensory booklet.” (Case 19)

“[The patient] would carry sensory items, and made use of low stimulus quiet areas.” (Case 6)

“[M]et prior to starting therapy to [help the patient] get accustomed to the therapeutic process, to the consulting room and for [the clinician] to adjust the consulting room accordingly (lights, window, fan and seating).” (Case 4)

#### Atypical eating behaviors

Cases presented with atypical eating behaviors, some caused by food-related sensory sensitivities and some by strict rules and routines around meals, which posed another challenge for the care team. Restricted food intake in EDs is typically connected to body image and fear of weight gain; however, in cases with co-occurring autism, food restriction could be due to other reasons such as the texture or smell of foods instead of the calorie content, or discomfort associated with swallowing or chewing, anxiety about eating with other people, and rigidity around timing of meals or the way food is prepared and served. In these cases, focusing on conventional targets for ED treatment, such as fear of weight gain, overlooks what could be the true cause of the atypical eating behaviors, creating barriers for patients' engaging in treatment.

“Atypical presentation- Enjoys calorie dense foods. … i.e., oat milk, mash potato, peaches, rice pudding and rice, chocolate and ice cream.” (Case 9)

“Food: small range at any time and then tires and stops eating them, resulting in the range of acceptable meals ever shrinking (This seems to be common within ASD patients).” (Case 16)

“At home, [the patient] eats just a small range of foods, eating the same foods repeatedly until [the patient] tires of them.” (Case 2)

“Highly anxious if something is presented differently than expected, i.e., crumbs falling off the Weetabix in [the patient's] bowl. … [Patient] has a preoccupation with numbers/measurements: i.e., precise measurements with fluid, weight, calories per day.” (Case 10)

Noticing the patients' atypical food preferences, dietitians developed an alternative menu (‘PEACE menu') that is calorie-matched to the standard menu on the ward but consists of more bland tasting food items that are more homogeneous in texture. Most items were also pre-packaged for consistency. Food experiments and gradual exposure to new food were also helpful for patients who struggled with unfamiliar foods.

“Menu choices: Repetitive, bland foods, colors, textures and flavors. …[the patient] has been utilizing the alternative menu a lot.” (Case 9)

“Has found it very useful to have the alternative menu choices.” (Case 15)

“For [the patient] to explore food, sniffing/touching without having to eat it, [the clinician] has offered [the patient] to explore/play with a few new things from the menu which [the patient] would like to try.” (Case 15)

#### Cognitive rigidity

Cognitive rigidity was also documented as a major challenge, particularly in terms of difficulty coping with setbacks and unpredictable changes in the environment, such as the sudden shift to the virtual setting owing to the Coronavirus pandemic. Patients who were more inflexible and rigid also tended to find it harder to break routines and showed more extreme emotional responses to such changes. Cognitive rigidity also made therapeutic engagement more difficult, as helping to push the patient toward change is often key to making progress.

“[The patient] keeps a precise idea in [their] head of what each thing should look like and cannot seem to settle until [the patient] can see exactly how the staff have measured [the patient's] food out.” (Case 10)

“Change is a huge source of anxiety. [The patient] depends on routines, sameness and predictability.” (Case 9)

“[The patient] struggles with engagement because of rigidity; very concrete [thinking style] which makes it difficult for [the patient] to relate the CRT (Cognitive Remediation Training) exercises to real life. [Patient] attributes this to autism and says [the patient] ‘is never going to change'.” (Case 13)

The team tried to help patients cope with changes by providing clear rationale for the plans. Patients were notified of any plans or potential changes early on to manage uncertainty. Most administrative changes were also made in consultation with patients.

“Most changes are collaborative. If major changes, the implementation is with some notice rather than straight away.” (Case 6)

“What works well: providing rationale for changes, boundaries in place, being clear on timeframes.” (Case 7)

Clinicians sometimes found that their own approach could be influenced by their patients' rigid way of behaving and inadvertently also become increasingly detail focused within their own practice. They were able to use case discussion as an opportunity for reflection and calibration of the team's approach.

“[The patient's] rigid way of behaving has led the team into becoming rigid and detail-focused as well, adding detailed conditions to [the patient's] passes just to avoid [the patient's] disruptive behaviors on the ward. The team will need to resist giving in to this and try to move to bigger picture and planning.” (Case 10)

#### Emotional difficulties

In addition to some cases displaying more extreme emotional reactions to changes, some had difficulty identifying and articulating their emotions during therapy sessions, leading to poorer therapeutic engagement. Clinicians found that this made planning and delivering therapy more challenging, as they had to speculate about the patient's feelings and the best ways to proceed with therapy with limited patient input.

“Perhaps [the patient] would agree to goals because I'd suggested them so sometimes it was tricky to work out what was meaningful to [the patient], especially as [the patient] didn't report having emotional responses to many things.” (Case 2)

“Emotions were not described well. ‘Don't know how to answer, not sure I can', ‘don't know how I feel'.” (Case 20)

The team therefore incorporated an emotions list into their practice in order to help patients to identify and express their emotions. In addition, a “traffic light communication system” was used to help patients to express both their emotions and the ways in which they wished to be supported to the clinical team.

“Developed Traffic Light Communication System for wider team. [Patient] had cards on bedroom door to indicate how [the patient] was feeling: Red = I am really struggling, approach me with the emotions list and ask me to mark what I am feeling; Amber = Today is difficult, check in on me and ask me how I am doing; Green = I am ok, everyone carry on as usual.” (Case 12)

## Discussion

This qualitative synthesis of case notes and PEACE huddle meetings provides a snapshot of the variety of challenges that clinicians face when treating complex patients with AN and autism, including communication difficulties, maintaining boundaries, issues related to autism screening, presence of comorbidities other than autism, sensory sensitivities, atypical eating behaviors, cognitive rigidity, and emotional difficulties.

### Helping patients with communication and emotional difficulties

Research has pointed out that one of the key problems for individuals with autism is communication in a social context, particularly with peers (31, 32). The case notes in this study further demonstrate how communication difficulties can, in practice, affect group participation as well as therapeutic engagement. Furthermore, patients' inability to maintain social relationships with peers can lead to over-dependence on carers (33) and clinical staff, creating a major barrier to independent living after discharge.

Clinicians in this study tried different resources and treatment adaptations for communication difficulties. One example was the ‘communication passport' (34), which is a one-page self-report document encompassing multiple aspects of communication, including a patient's preferred way to communicate, their sensory needs, their dislikes and their special interests and strengths. This worksheet was designed to help health professionals understand patients' preferred ways of communicating. Another adaptation described in the case notes was individual-level modification of communication style. For patients who struggled with open ended questions, which is not unusual in individuals with autism (35), multiple choice questions were sometimes used as an alternative.

Additionally, patients often found it challenging both to identify and to articulate their emotions. However, it should be noted that emotional difficulties are widely present in the overall ED population, rather than limited to those with autism comorbidity. Indeed, there is a large body of existing work on alexithymia in patients with ED (36) as well as autistic individuals (37). Thus, the adaptations and resources used to address emotional difficulties may be helpful to all patients with ED, with or without co-occurring autism. To help patients identify their emotions, Cognitive Remediation and Emotion Skills Training (CREST) (38) was delivered in both individual and group formats. CREST interventions have been shown to significantly improve alexithymia and motivation in patients with AN and autism (39). On the other hand, patients with difficulties articulating their thoughts and emotions were given options to use conversation cards or ‘traffic light' communication system to indicate their emotions, or to represent their thoughts through art or diagrams instead. These methods received good feedback in several cases, but their validity should be explored further in future research.

### Boundary maintenance in adapting treatment for autism and other comorbidities

Previous studies have discussed boundary crossings, which are defined as attempts to “adapt an existing therapeutic alliance to foster the patient's capacity to work in therapy” (40). Boundary crossings are usually benign modifications to accommodate reasonable requests and individualize treatment. They become problematic when there is a negative impact on patients, endangering their health, independence, and/or recovery (41). In the setting of this study, clinicians were highly attuned to the different needs of patients with the comorbidity and were open and prepared to make adaptations. As a result, difficulties maintaining rules and boundaries spanned most of the case notes reviewed. Clinicians in this study often found themselves facing the dilemma of whether to continue encouraging change in patient behavior for recovery from ED, or to make accommodations for autism-specific needs.

Rather than adhering rigidly to absolute boundaries in all situations, clinicians often endeavored to compromise with patients. Furthermore, they worked with patients collaboratively to investigate what was driving the presenting difficulty before deciding whether treatment boundaries could be adapted: whether it was an ED symptom that should be addressed, an autism-driven need that could be accommodated, or an autism-related difficulty that nevertheless should be managed to facilitate independent living. Clarifying the cause of patients' problems was a crucial step to developing a corresponding care plan. Previous research on a framework for differentiating between clinical features of autism and ED could be a useful guiding tool for clinicians facing similar dilemmas (6). In some cases, however, comorbidities other than autism (e.g., anxiety, OCD and EUPD) were also present and intertwined with autism and the ED. This is consistent with existing evidence of overlap between EUPD and autism (42) and OCD and autism (43). These comorbidities and ED often fuelled each other by contributing to similar patterns of thoughts and behaviors, making recovery even more difficult. Therefore, more work may be required for clinicians to differentiate between the symptoms and identify the best way to help patients. This suggests the need for an extensive guiding framework for differentiating between difficulties caused by ED, autism, and other common comorbidities such as OCD and EUPD.

### Sensory difficulties, cognitive rigidity and atypical eating behaviors

Both sensory difficulties and cognitive rigidity were linked to atypical eating behaviors in people with AN and autism, suggesting that their presentation may be driven by autism-related sensory and cognitive difficulties rather than common ED symptomatology such as fear of weight gain. Adaptations were necessary in these cases since conventional treatment at the ED services targeted typical ED symptoms and ED-driven cognition. In some cases, patients' preferences for certain foods were based on the texture, temperature, or even color, instead of calorie content (e.g., preference for smooth-textured, high calorie foods like ice cream). Weight restoration, therefore, could be easier for these patients once a sensory friendly dietetic plan was in place, since their primary concern was not weight or body shape. This is consistent with previous research showing that inpatients with AN and high autistic characteristics showed more improvement in Body Mass Index (BMI) after treatment than peers without autism (44).

In some cases, patients also had rigid rules around eating and could be exceedingly selective, such as limiting intake to a few categories of foods or only eating pre-packaged food. Clinicians found food experiments and gradual exposure to novel foods helpful when patients presented with selective eating behavior. Such interventions are mostly used with avoidant/restrictive food intake disorder (ARFID) and aim to reduce anxiety related to food and eating, and the extent of food neophobia (45). People with autism share a similar presentation to people with ARFID, including a preference for familiar foods and an aversion to trying new things (46), which inspired the team to try food experiments with the patients. Recent research has also found that fussy eating partially mediates the associations between autism and the development of ED behaviors (47), suggesting that fussy eating may be a useful point for prevention and intervention.

One challenge, however, with introducing food experiments to an ED service, was its initial contradiction with usual practice where patients were expected to finish their meals instead of playing with food without eating it. Extensive team-wide discussions were held before all clinical staff reached consensus on which patients could utilize the food experiments and for how many sessions. The costs and resources required to deliver the intervention also need to be considered before food experiments can be made regular practice. Overall, although food experiments were found to be helpful with some of the cases with autism comorbidity, this is not yet validated and therefore warrants future testing.

### Need for pragmatic autism screening tools suitable for ED services

This study also highlights a need for a pragmatic autism screening approach in ED services. The AQ-10 was used in the service for its brief format and convenience, and has the advantage of being a screening instrument to identify individuals who would benefit from a full autism assessment (9). However, its validity and reliability for use with this specific patient population are yet to be tested. Indeed, in some cases, patients previously had a formal autism diagnosis or deemed by clinicians to have a strong autistic presentation that would benefit from treatment adaptations, but still scored below the threshold on the AQ-10. Furthermore, it is still unknown whether the AQ-10 is specific enough to differentiate between autism and other common comorbidities such as social anxiety, given that certain items on the AQ-10 may tap into symptoms of social anxiety rather than autism. Clinical practice would benefit from future research focusing on pragmatic screening tools with higher specificity and sensitivity when used in this co-morbid population. Combined use of the AQ-10 with other self-report screening measures for increased validity, such as sensory sensitivity screening (48) or more detailed self-report measures like the Social Responsiveness Scale (28), should also be considered.

### Limitations

This study focused on a relatively small number of cases and only half of the cases had formal diagnosis of autism, therefore, the findings of this case synthesis cannot be generalized to the wider population of patients with comorbid autism and ED. However, the clinical reality and challenges raised by the clinicians in this study provide important learnings for future treatment improvement and adaptations, as well as future research. The lack of a suitable autism screening tool was also noted for this clinical group. Future research should consider incorporating more valid screening tools, such as the longer version of AQ (49), the Camouflaging Autistic Traits Questionnaire [CAT-Q; (50)], or the Sensory Processing Measure, Second Edition [SPM-2; (51)] that investigate more of the behavioral aspects in this population. Furthermore, as the cases concerned were de-identified, it was not possible to trace the patients' clinical records to identify outcomes such as BMI improvement, limiting the range of reportable measures to complement clinicians' reports of what was helpful. However, ongoing evaluation of the PEACE Pathway will provide evidence on effectiveness to support its wider implementation.

## Conclusions

Clinicians face a variety of challenges when providing care to patients with comorbid ED and autism, including dealing with communication difficulties, boundary issues, problems with autism screening, managing and differentiating comorbidities, sensory difficulties, atypical presentation of eating behaviors, cognitive rigidity, and emotional difficulties. The exploratory findings of this synthesis serve to generate hypotheses for future investigation to identify ways in which health professionals can address these difficulties and develop protocols for dealing with clinical dilemmas in adapting treatment.

## Data availability statement

The data analyzed in this study is subject to the following licenses/restrictions: The original case notes are not available in order to protect patient privacy. The summarized datasets generated for this study are available from the corresponding author on reasonable request. Requests to access these datasets should be directed to zhuo.li@kcl.ac.uk.

## Ethics statement

The studies involving human participants were reviewed and approved by the Clinical Governance and Audit Committee at South London and Maudsley NHS Trust. Written informed consent for participation was not required for this study in accordance with the national legislation and the institutional requirements.

## Author contributions

ZL analyzed, interpreted the data, and was a major contributor in producing the manuscript. CH-H co-analyzed data and contributed to editing of the manuscript. KT is a principal investigator of the PEACE pathway project and conceived of the presented study idea. KT and SB co-supervised the study and contributed to the drafting of the manuscript and interpretation of results. All authors read and approved the final manuscript.

## Funding

KT received funding from the Maudsley Charity, an independent NHS mental health charity which works in partnership with patients and families, clinical care teams, and researchers at South London and Maudsley NHS Foundation Trust. KT also received funding from MRC-MRF Fund (MR/S020381/1; BiomaRkers for AnorexIa NErvosa and autism spectrum Disorders- longitudinal study).

## Conflict of interest

The authors declare that the research was conducted in the absence of any commercial or financial relationships that could be construed as a potential conflict of interest. The reviewers UF and HH declared a shared affiliation with the authors ZL, SB, and KT to the handling editor at the time of review.

## Publisher's note

All claims expressed in this article are solely those of the authors and do not necessarily represent those of their affiliated organizations, or those of the publisher, the editors and the reviewers. Any product that may be evaluated in this article, or claim that may be made by its manufacturer, is not guaranteed or endorsed by the publisher.
